# Guided cracking of electrodes by stretching prism-patterned membrane electrode assemblies for high-performance fuel cells

**DOI:** 10.1038/s41598-018-19861-6

**Published:** 2018-01-19

**Authors:** Chi-Yeong Ahn, Segeun Jang, Yong-Hun Cho, Jiwoo Choi, Sungjun Kim, Sang Moon Kim, Yung-Eun Sung, Mansoo Choi

**Affiliations:** 1 Center for Nanoparticle Research, Institute for Basic Science (IBS), Seoul, 08826 Korea; 2 School of Chemical and Biological Engineering, Seoul National University, Seoul, 08826 Korea; 3 Global Frontier Center for Multiscale Energy Systems, Seoul National University, Seoul, 08826 Korea; 4 Department of Mechanical and Aerospace Engineering, Seoul National University, Seoul, 08826 Korea; 5 Department of Chemical Engineering, Kangwon National University, Samcheok, 25913 Korea; 6 Department of Mechanical Engineering, Incheon National University, Incheon, 22012 Korea

## Abstract

Guided cracks were successfully generated in an electrode using the concentrated surface stress of a prism-patterned Nafion membrane. An electrode with guided cracks was formed by stretching the catalyst-coated Nafion membrane. The morphological features of the stretched membrane electrode assembly (MEA) were investigated with respect to variation in the prism pattern dimension (prism pitches of 20 μm and 50 μm) and applied strain (*S* ≈ 0.5 and 1.0). The behaviour of water on the surface of the cracked electrode was examined using environmental scanning electron microscopy. Guided cracks in the electrode layer were shown to be efficient water reservoirs and liquid water passages. The MEAs with and without guided cracks were incorporated into fuel cells, and electrochemical measurements were conducted. As expected, all MEAs with guided cracks exhibited better performance than conventional MEAs, mainly because of the improved water transport.

## Introduction

Polymer electrolyte membrane fuel cells (PEMFCs) have received significant attention over the past several decades as future clean energy devices because they do not emit pollutants and they provide high energy conversion efficiencies^1–7^. Although many technological advances have been achieved in the research field of PEMFCs, some practical issues still hinder their commercialization. To obtain high device performance, ohmic loss should be reduced by using a thinned electrolyte membrane^8–10^ and water transport at the cathode of the membrane electrode assembly (MEA) should be enhanced^11–14^. In relation to water transport, water molecules that are generated by the oxygen reduction reaction block the catalyst surface and pores in the cathode catalyst layer, reducing device performance. Hence, the produced water must be appropriately removed to maintain the pathway for the reactant oxygen gas. Many attempts have been made to effectively remove the water generated by the electrochemical reaction at the cathode. First, hydrophobic polymer nanoparticles, such as polytetrafluoroethylene, were mixed with a catalyst ink and inserted into the cathode catalyst layer during the MEA fabrication process^15–17^. However, the inserted hydrophobic polymers adhere to the carbon support and catalyst surface without selectivity, which decreases the electrochemically active surface area of the catalyst layer. Another method to improve water transport is to introduce pore-forming agents, such as carbonate or polystyrene particles, which are removed after the catalyst layer is sprayed onto the electrolyte membrane^18–20^. These methods require chemical or thermal post-treatments, which are complex and time consuming.

Our recent study showed that cracks generated on the cathode (formed by stretching the MEA) simultaneously enhanced mass transport and reduced membrane resistance^10^. This simple and facile method does not require any post-treatment after stretching the MEA, and the cracks on the electrode effectively act as macropores to improve mass transport. However, the effects of the crack size and the areal fraction of cracks on device performance have not been studied, and reproduction of the same crack morphology is not easy in the case of MEAs with randomly distributed cracks. To address these issues, we deliberately generated controlled cracks using the concentrated surface stress^20–24^ of a prism-patterned membrane by varying the pattern size and the applied strain. Further, we directly observed that the cracks in MEAs acted as water reservoirs and water passage openings using environmental scanning electron microscopy (ESEM). To assess the effect of cracks with different width and areal fractions on fuel cell device performance, we conducted various electrochemical studies by comparing the device performance of MEAs with and without cracks. All the MEAs with guided cracks exhibited better performance than the conventional MEA, mainly because of the improved water transport.

## Results

### Generation of guided cracks in the cathode

**Figure 1:**
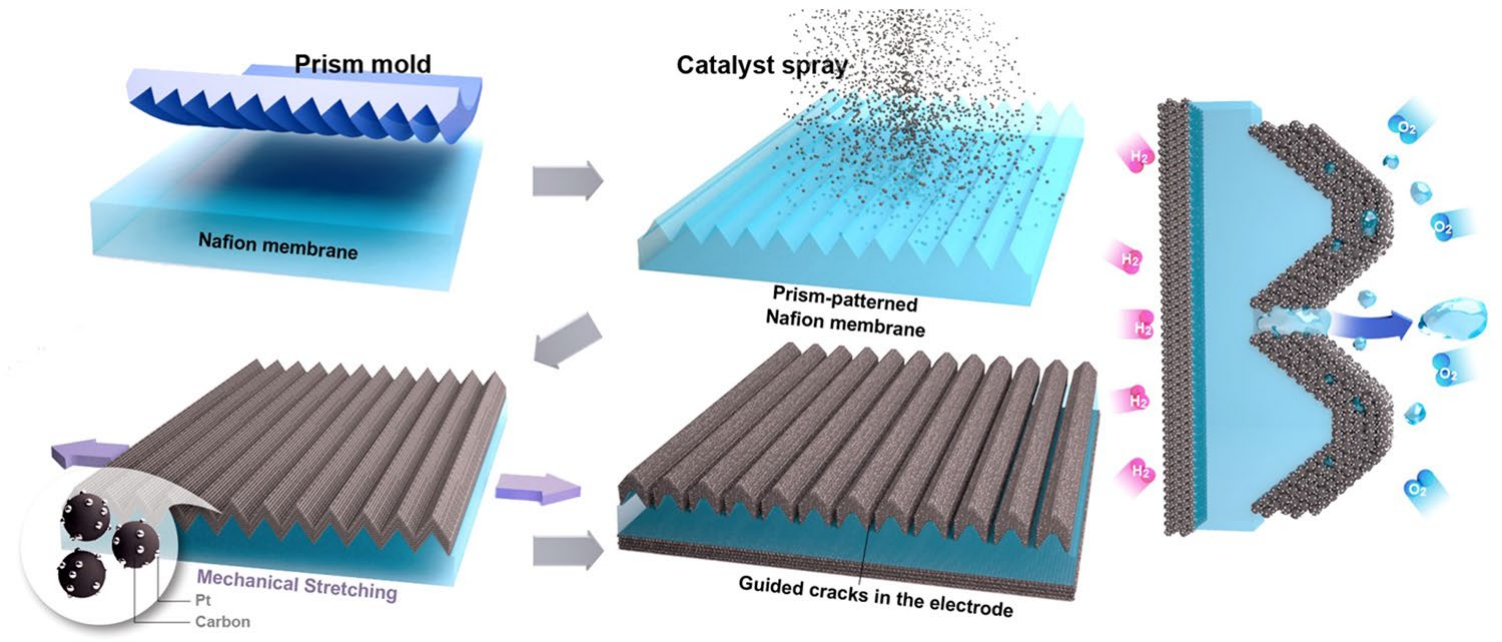
Schematic illustration of guided crack generation in an electrode by stretching the prism-patterned membrane electrode assembly.

Microprism-patterned arrays consist of repeated valleys and ridges. When the elastic prism structure is stretched in the direction perpendicular to the prism-line direction, the stress is concentrated in the valleys of the prism pattern and the structures are deformed, as shown in Supplementary Figure S1. This means that we can produce confined surface stress in a specific region by controlling the surface structure and applying stress. When rigid material-coated soft prism patterns are stretched, cracks are generated at the interface between the two attached surfaces because of the elastic modulus mismatch when the surfaces are stretched or pressed^25,26^. Additionally, cracks are generated in the valleys of the prism patterns. Figure 1 shows a schematic illustration of the generation of guided cracks in a porous electrode composed of carbon-supported Pt catalysts (Pt/C). Initially, microprism patterns are carved onto a Nafion 212 membrane (thickness ~50 μm) by thermal imprinting. Then, catalyst ink is sprayed onto the prism-patterned Nafion membrane to construct a porous electrode at the cathode side. When the catalyst-coated Nafion membrane is stretched, the different elastic modulus values of the Nafion membrane and the catalyst layer induce cracking in the catalyst layer. If the applied strain (ε), which is defined as the change in length after stretching divided by the original length (ε = ∆L/L), extends beyond the elastic region, the membrane does not fully recover to its original length; this phenomenon is called plastic deformation. Then, cracks between catalyst islands are generated. The crack width increases as the strain applied to the catalyst-coated membrane increases. In our experiments, we concentrated stress in the valleys of the prism patterns and controlled the stress distribution by varying the pattern pitch size. As a result, line-shaped cracks were generated in the valleys with specific lengths.

### Physical properties of the prism-patterned Nafion membrane

**Figure 2:**
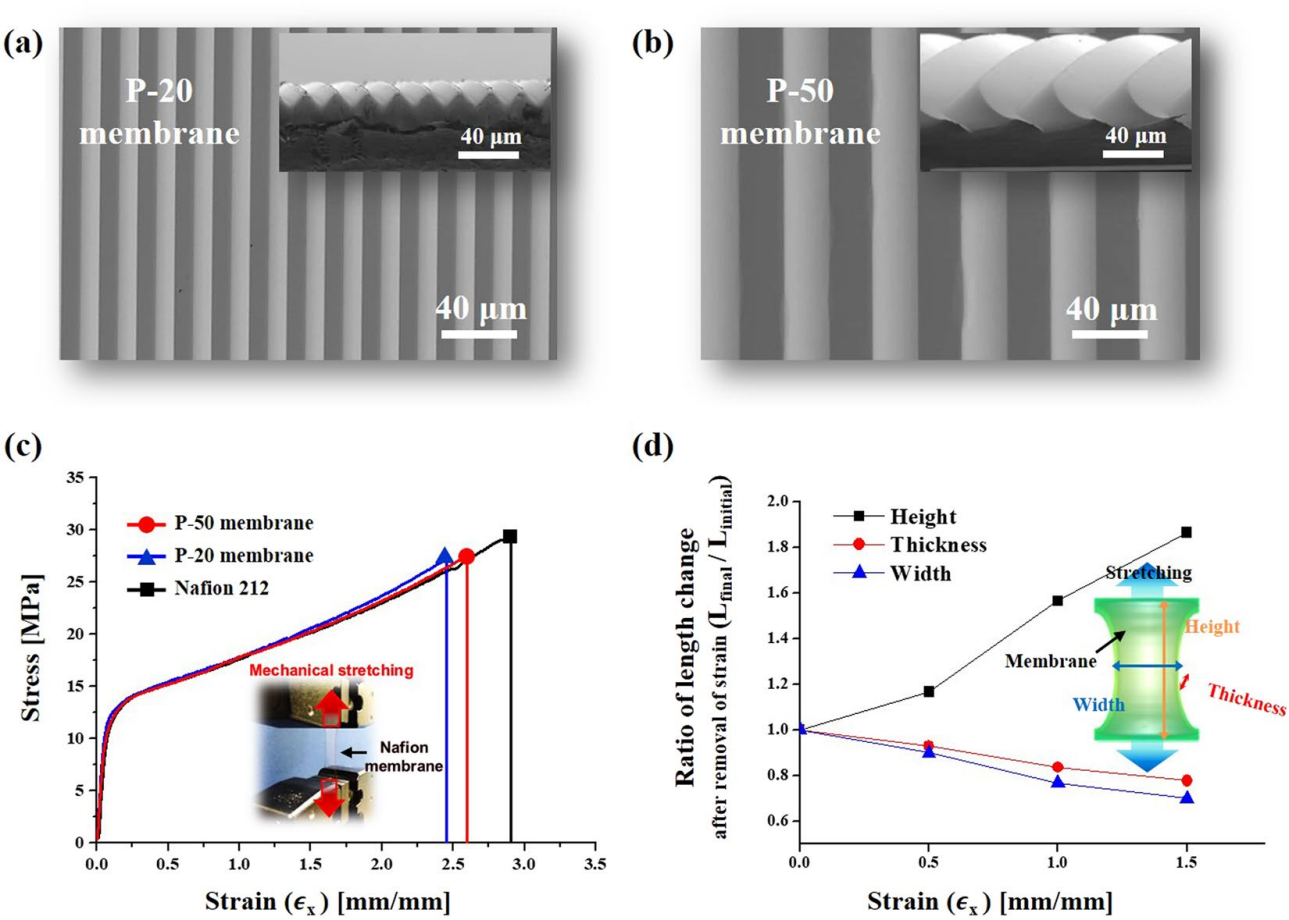
(**a**,**b**) SEM images of a Nafion membrane imprinted with a prism mould with a pitch of 20 μm (P-20 membrane) (**a**) and 50 μm (P-50 membrane) (**b**). (**c**,**d**) Stretching properties of the Nafion membranes: (**c**) strain–stress curves of the Nafion 212 membrane, 20 μm prism-patterned Nafion membrane, and 50 μm prism-patterned Nafion membrane measured by applying strain to the membranes (Inset: optical image showing the operation of the stretching machine); (**d**) ratio of height, width, and thickness changes of the Nafion membranes with varying strains (~0.5, ~1.0, and ~1.5).

To investigate the mechanical properties of prism-patterned Nafion membranes, we conducted uniaxial tensile strength tests on a pristine Nafion 212 membrane (thickness of ~50 μm) and Nafion membranes with microprism patterns with a 20 μm pitch (P-20 membrane) and a 50 μm pitch (P-50 membrane). Figure 2a and b show that the two prism patterns have the same angle (~45°). Figure 2c shows the stress–strain curves of the prepared membranes. The pristine Nafion 212 membrane shows an elongation to break of ~291% and a maximum tensile strength of ~29.48 MPa. Thus, the Nafion 212 membrane can be elongated by a factor of three with respect to its original length. In the case of the prism-patterned Nafion membranes, the P-20 membrane is stretched to ~260% and the P-50 membrane is stretched to ~245% (until breakage, Supplementary Table S1). The elongation to break values are lower for the prism-patterned Nafion membranes than for the pristine Nafion membrane; however, the prism-patterned membranes still exhibit high elongation properties despite the existence of valleys where the stress is focused. The strain–stress curves for the three cases show that the membranes become plastically deformable when the applied strain is above ~0.1 (~10% elongation). This means that the membranes undergo permanent deformation (incomplete recovery to the original length) in the strain region from a strain value of ~0.1 to the break points. In this study, we used a broad plastic deformation region to generate cracks and controlled the crack size by varying the strain (0.5 and 1.0). Figure 2d shows the relation between the applied strain and the ratio of altered lengths (height, thickness, and width) after removing the applied strains (0.5, 1.0, and 1.5). The height refers to the length of the membrane in the stretching direction, and the width is the length in the direction orthogonal to the stretching direction. Generally, when a specific material is stretched, the length of the specimen elongates in the stretching direction, whereas the length in the orthogonal direction decreases. To use MEAs with cracked electrodes in square single cells, we sprayed a catalyst layer onto rectangular-shaped membranes, considering the length changes of the membranes during stretching.

### Morphological features of the generated guided cracks with variation of the strain

**Figure 3:**
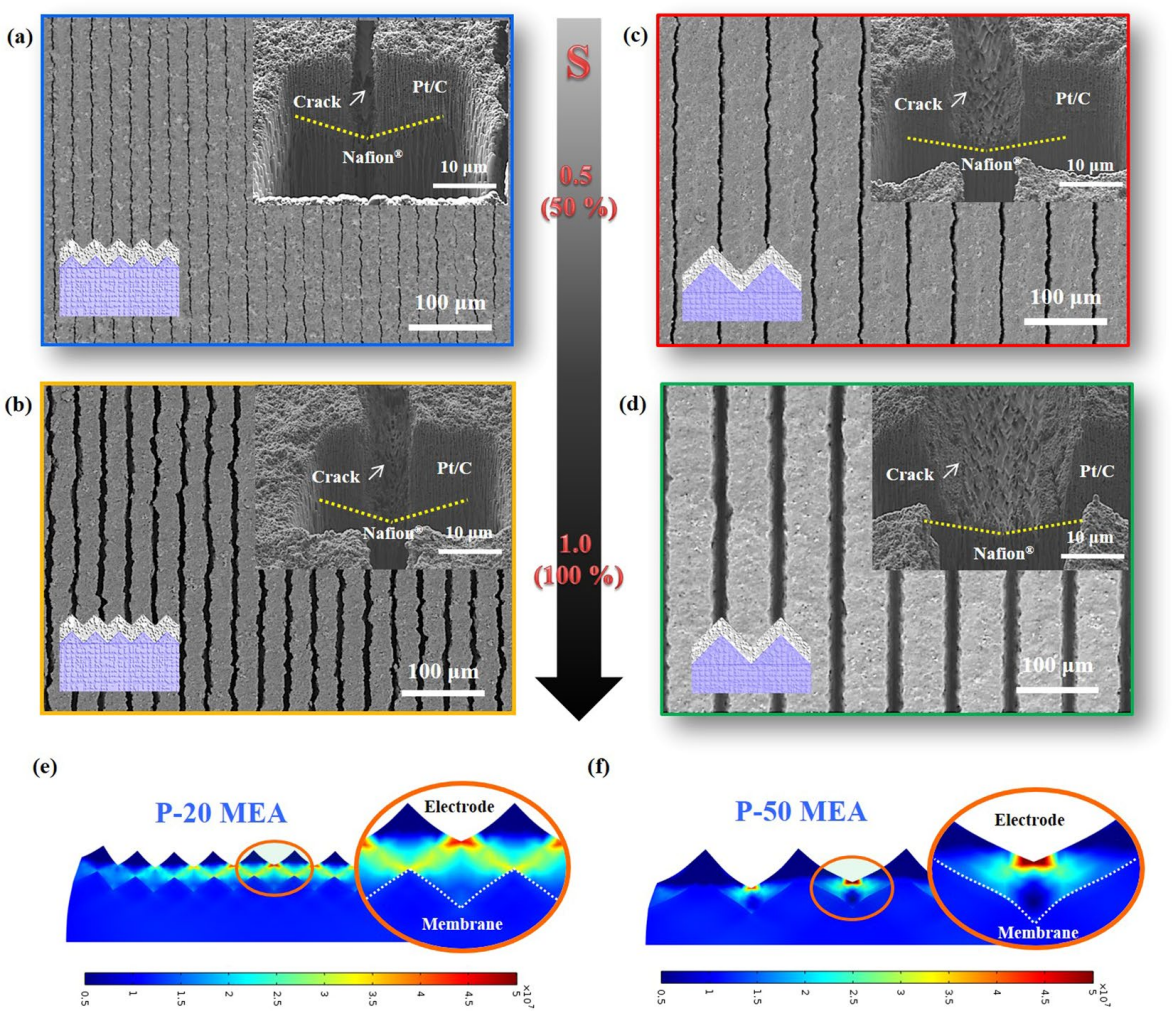
Morphological features of the generated guided cracks: (**a**–**d**) SEM images of the cracks generated after applying strains (*S*) of ~0.5 and ~1.0 to the prism-patterned MEAs with pitches of 20 μm and 50 μm: (**a**) P-20 MEA with *S* ≈ 0.5, (**b**) P-20 MEA with *S* ≈ 1.0, (**c**) P-50 MEA with *S* ≈ 0.5, and (**d**) P-50 MEA with *S* ≈ 1.0. (**e**,**f**) Simulations of the stress distributions on the prism-patterned MEAs with pitches of 20 μm (**e**) and 50 μm (**f**).

To elucidate the geometrical features of the generated cracks in the electrodes on prism patterns, we applied strains (0.5 and 1.0) to the MEAs and observed the morphological changes of the cracks using SEM (Fig. 3a–d). Straight cracks were formed in the prism-line direction for the P-20 and P-50 MEAs. The straight cracks in the electrode were generated by stress concentration in the valley of the prism patterns. The finite elements method (FEM) simulation confirmed that the stresses caused by stretching the MEAs are focused on the electrode surfaces (Fig. 3e and f). However, for a prism pattern with a pitch of ~10 μm (P-10) (Supplementary Figure S2), the formed cracks were randomly distributed compared with those for the P-50 and P-20 MEAs. There are two main reasons for the incomplete guided cracks in the P-10 MEA. First, the stresses are not fully concentrated in the valleys because of the low height (proportional to pitch) feature of P-10 relative to the electrode thickness. Second, when applying the same magnitude of stress (strain), the stress is more geometrically distributed on the P-10 MEA because the number of valleys on the P-10 MEA is five and two times larger than those on the P-50 and P-20 MEAs, respectively. These results imply that we should simultaneously consider the pitch size of the prism pattern and the electrode thickness to generate well-guided cracks.

To investigate the morphological features of the fully guided cracks of the P-20 and P-50 MEAs, we analysed the corresponding SEM images for each strain and pattern dimension. For an applied strain of ~0.5, the average crack width in the P-20 MEA was ~2.1 μm and that in the P-50 MEA was ~5.3 μm. Further, the areal fractions of the two samples were almost the same (~8.0%). When the applied strain was increased to ~1.0, the crack widths were enlarged. The average width in the P-20 MEA was ~8.4 μm and that in the P-50 MEA was ~21 μm, with areal fractions of 19.42%. The average width is proportional to the pattern dimension (pitch of the prism pattern), and the areal fraction of cracks increases proportionally as the applied strain increases. With the same amount of catalyst loading, the thickness of the electrode increases as the areal fraction of the cracks increases, as confirmed by observing cross-sectional SEM images (Supplementary Figure S3). As the applied strain increased, the area of exposed bare Nafion surface increased and this resulted in decrease of areal fraction of the electrode area. In our experiments, we used the same amount of catalyst and thus the electrode thickness increased by ~14% and ~24% for strains of ~0.5 and ~1.0, respectively.

### Behavior of water on the cracked electrode

**Figure 4:**
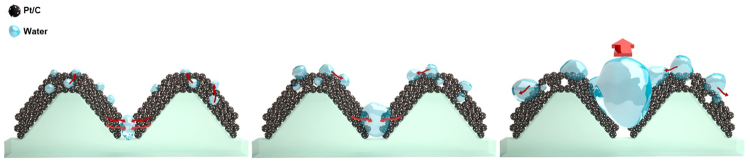
Schematic illustrations of the behaviour of generated water in the controlled cracks in the catalyst layer.

**Figure 5:**
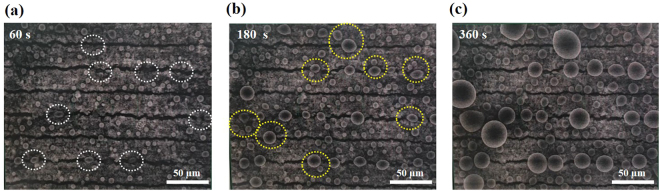
ESEM images of water behaviour on a 20 µm cracked catalyst layer (strain ≈ 0.5) with respect to time: (**a**) 60 s, (**b**) 180 s, and (**c**) 360 s. The chamber pressure and substrate temperature were maintained at ~5.1 Torr and ~2 °C, respectively (RH = 95%).

Figure 4 shows schematic illustrations of how generated water behaves in the controlled cracks in the catalyst layer. The water behaviour can be divided into three major steps. First, the generated water inside the catalyst layer moves to the surface of the catalyst layer and the cracks. Next, water droplets form and grow by coalescing with neighbouring drops and the cracks fill with liquid water. After filling the cracks in the electrode, droplets arise from the cracks and become larger by coalescing with neighbouring droplets, and are finally ejected to the gas diffusion layer. To visually demonstrate the role of the cracked electrode, we directly observed the water behaviour on the surface of the cracked electrode using *in-situ* ESEM (Fig. 5). When the RH reaches 95%, a liquid water droplet forms and the cracks fill with liquid water (white dotted circles in Fig. 5a) within 1 min. After approximately 180 s, droplets arise from the cracks (yellow dotted circles in Fig. 5b) and grow by coalescing with neighbouring droplets (Fig. 5c and movie clip available). From these observations, we confirmed that the guided cracks in the electrodes act as water reservoirs and also as water passages. These properties are desirable for enhancing fuel cell performance by reducing mass transport resistance.

### Enhanced performance using cracked MEAs in PEMFCs

**Figure 6:**
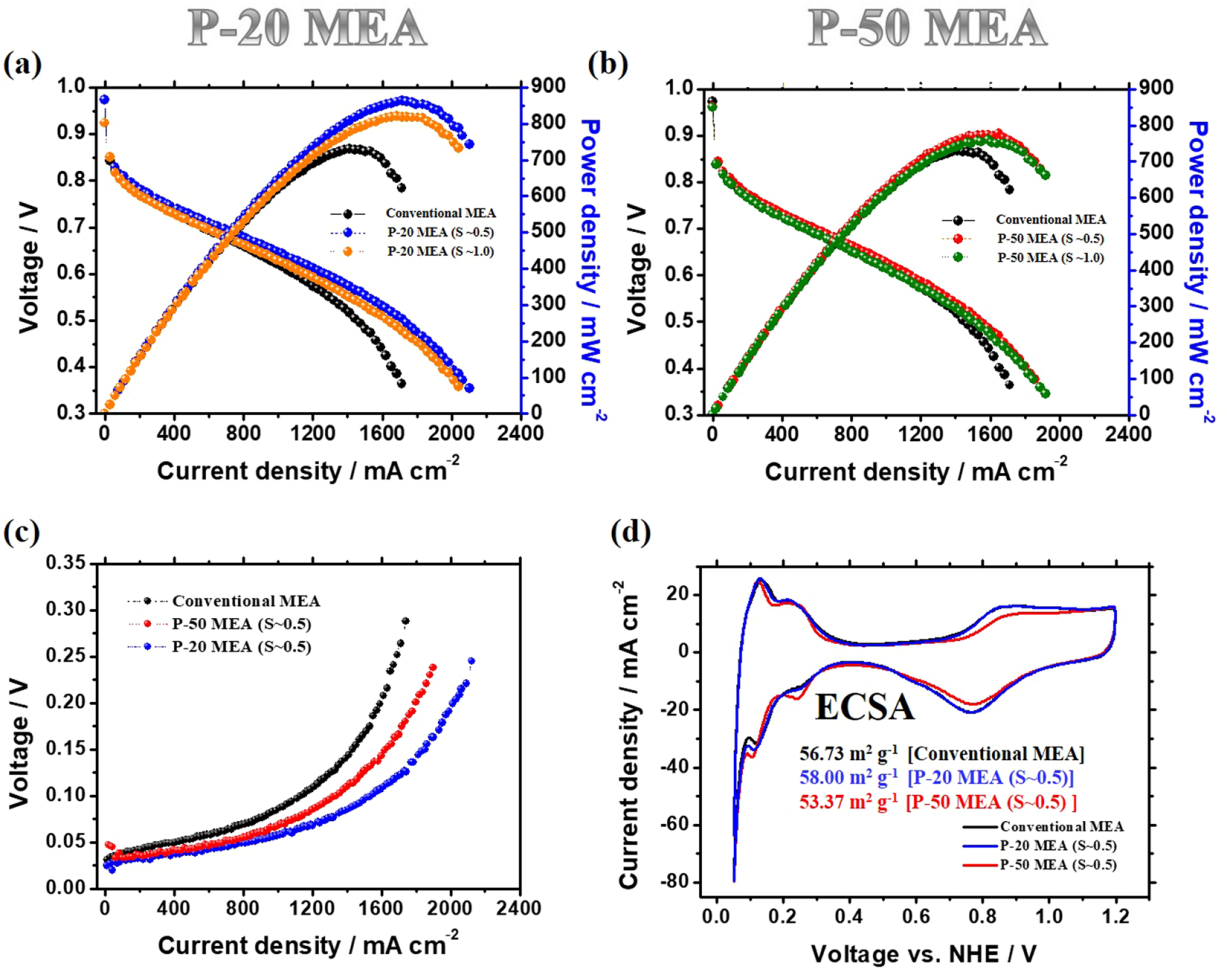
**Electrochemical measurements of** the MEAs: (**a**,**b**) Polarization curves of a conventional MEA and the MEAs with cracks generated using a 20 μm prism pattern (*a*) and a 50 μm prism pattern (**b**) with various strains (~0.5 and ~1.0) under H_2_/air. (**c**) Oxygen gains obtained under ambient pressure. (**d**) Cyclic voltammetry (CV) measurements of the prism-patterned MEAs with the same strain (~0.5) and a conventional MEA at the cathode catalyst layers (NHE = normal hydrogen electrode).

To elucidate the effect of the guided cracks on PEMFC performance, we constructed single cells by incorporating guided cracked MEAs. The single cells with guided cracked MEAs were operated by flowing fully humidified H_2_/air to the anode/cathode. The experimental sets were composed of P-20 and P-50 MEAs with applied strains of ~0.5 and ~1.0, respectively. These MEAs exhibit enhanced performance mainly because of the improved water transport under all conditions, regardless of the dimension of the prism pattern and the applied strain, relative to that of the conventional MEA (Fig. 6 and Supplementary Figure S4). The performance improvement of the P-20 MEA is higher than that of the P-50 MEA with the same applied strain (*S* ≈ 0.5 and 1.0). Considering that the same applied strain indicates an analogous areal fraction of generated cracks, improved performance can be obtained by increasing the number of guided cracks. The performance with an applied strain of ~1.0 is lower than that with an applied strain of ~0.5 for the P-20 and P-50 MEAs. The MEAs with *S* ≈ 0.5 show higher performance than the MEAs with *S* ≈ 1.0, which can be explained by the morphological features of the stretched MEA. Generally, the interface between the Nafion membrane and the electrode is the site where the electrochemical reaction is most effective^27^. However, as more strain was applied to the MEA, the area of the bare Nafion membrane surface (without contact with the catalyst layer) increased, which reduced the electrochemical reaction and reduced the device performance for the MEAs with *S* ≈ 1.0. In addition, as mentioned above, MEAs with *S* ≈ 1.0 have thicker electrodes than *S* ≈ 0.5, which could also lead to reduced performance. These results indicate that there exists a desirable crack density and applied strain to obtain a high-performance MEA. In our experiments, the P-20 MEA with an applied strain of ~0.5 shows the highest performance, with a maximum power density of ~863 mW cm^−2^ and a current density of ~1,272 mA cm^−2^ at 0.6 V under H_2_/air. This maximum power density is ~18% higher than the conventional value (~730 mW cm^−2^) (Supplementary Figure S2).

To further confirm the degree of mass transfer at the cathode catalyst, we calculated the oxygen gain (Δ*V*) using the following equation^14,28^:

1 $${\rm{\Delta }}V={V}_{({H}_{2}/{O}_{2})}-{V}_{({H}_{2}/air)}$$

The oxygen gain values of the MEAs with guided cracks are smaller than that of the reference MEA, and the difference increases as the current density increases (Fig. 6c). The P-20 MEA (*S* ≈ 0.5) shows the highest performance among the prepared MEAs, which implies that the guided cracks in the MEA enhance mass transport relative to that in the conventional MEA. We also measured the device performance of an MEA using a pre-stretched membrane while excluding the thinning effect of the stretched membrane, as shown in Supplementary Figure S5. The prism-patterned Nafion membrane was previously stretched (P-20 membrane with *S* ≈ 0.5) before spraying the catalyst. Then, the catalyst ink was sprayed onto the pre-stretched membrane to construct the MEA. In this manner, the MEA with a pre-stretched membrane was prepared with no cracks in the cathode but it contained a thinned membrane. The performance of the MEA with the pre-stretched membrane is slightly higher than that of the conventional MEA. This is because the vertically asymmetric geometrical feature at the interface between Nafion membrane and catalyst layer lead to improved water transport^28^. However, it showed still much lower performance than that of the P-20 MEA with guided cracks under H_2_/air, which indicates that the guided cracks has an effective role to enhance the device performance.

### Electrochemical analysis of the MEA with guided cracks

**Figure 7:**
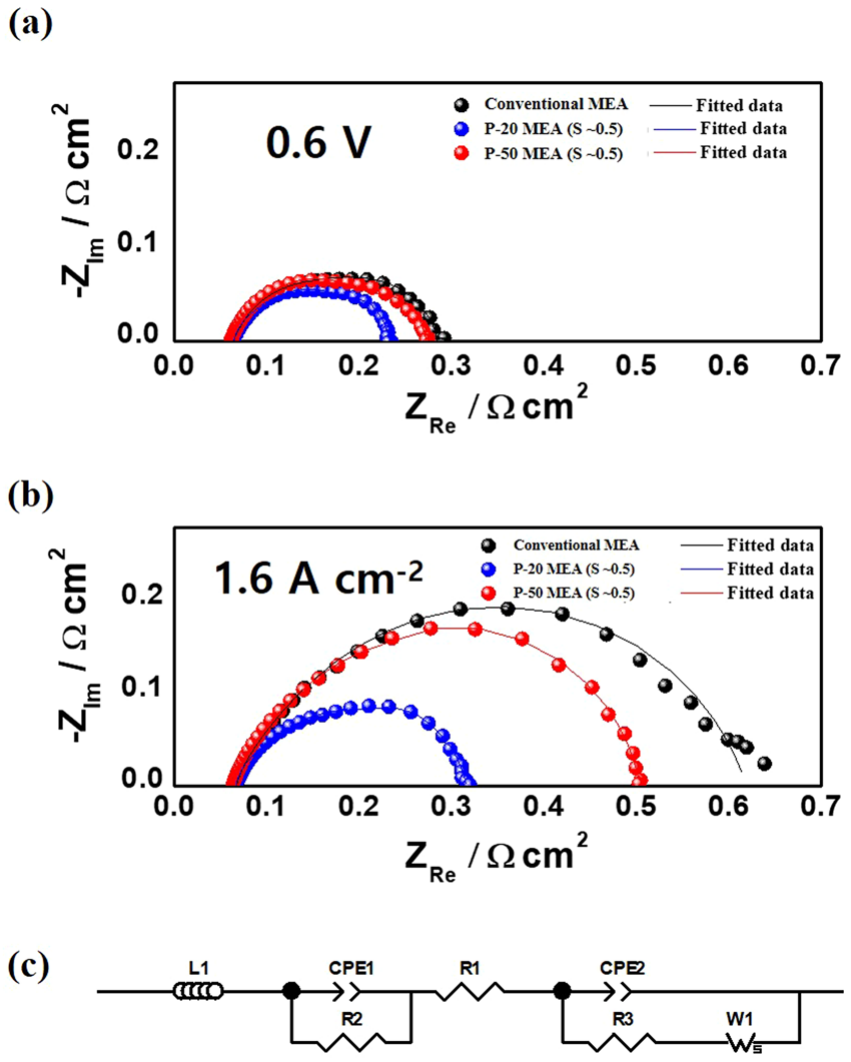
Electrochemical impedance spectroscopy (EIS) measurements of prism-patterned MEAs with the same strain (~0.5) and a conventional MEA at 0.6 V vs. a reversible hydrogen electrode (**a**) and at 1.6 A cm^−2^ (**b**). (**c**) Equivalent circuit of the PEMFC (L1 = inductance of the electric wire, R1 = internal membrane resistance, R3 (2) = charge transfer resistance of the cathode (anode), CPE2 (1) = constant phase element of the cathode (anode), and W1 = Warburg impedance).

To quantitatively investigate the effects of enhanced water transport, EIS and CV were performed for the P-20 and P-50 MEAs with an applied strain of ~0.5 and compared with the respective data for a conventional MEA (Figs 6d and 7). Electrochemical active surface areas (ECSAs) were obtained from the CV measurements^29^. The measured ECSAs of the conventional MEA (56.73 m^2^ g^−1^), P-20 MEA with *S* ≈ 0.5 (58.00 m^2^ g^−1^), and P-50 MEA with *S* ≈ 0.5 (53.37 m^2^ g^−1^) are comparable, which shows that the area of the tri-phase boundary is barely affected by the guided cracks. The EIS data for the three samples were obtained at 0.6 V and fitted to the equivalent circuit, as illustrated in Fig. 7^30–32^. The P-20 and P-50 MEAs with *S* ≈ 0.5 show comparable ohmic resistances of 0.0637 Ω cm^2^ and 0.0609 Ω cm^2^, respectively (that of the conventional MEA is 0.0631 Ω cm^2^). Even though the current densities of the cracked MEAs are higher than that of the conventional MEA at 0.6 V, the Warburg impedance values of the P-20 MEA (0.02026 Ω cm^2^) and P-50 MEA (0.03817 Ω cm^2^) with *S* ≈ 0.5 are much smaller than that of the conventional MEA (0.09565 Ω cm^2^) (Supplementary Table S3). Moreover, in the high current density region (~1.6 A cm^−2^), the difference between the Warburg impedance values for the P-20 MEA with *S* ≈ 0.5 (0.0326 Ω cm^2^) and the conventional MEA (0.3702 Ω cm^2^) increases (Supplementary Table S3 and Fig. 7b). These results imply that water transport at the cathode catalyst layer is substantially improved by the cracks, especially for P-20 MEA, providing high device performance.

## Discussion

In this study, we achieved enhanced device performance by incorporating guided cracks into a porous electrode by stretching the catalyst-coated membrane to improve water transport. We successfully generated guided cracks using the concentrated surface stress in an electrode with a prism-patterned Nafion membrane. We investigated the effects of crack size and the areal fraction of cracks on device performance by varying the dimension of the pattern (prism pitches of 20 μm and 50 μm) and the applied strain (*S* ≈ 0.5 and 1.0). We found that the guided cracks in the electrodes have effective roles as generated water reservoirs and as water passages by observing the behaviour of the generated water in the guided and cracked electrodes using *in-situ* ESEM. We incorporated MEAs with guided cracks into single cells to examine the electrochemical performance. Under all conditions (regardless of the prism pattern dimension and applied strain), the MEAs with controlled cracks exhibited improved performance. In particular, the maximum power density of P-20 MEA (with an applied strain of ~0.5) increased by ~18% relative to that of the conventional MEA. The guided cracks in the electrode effectively enhanced water transport on the cathode side, as confirmed by EIS measurements. Our strategy to generate controlled voids, i.e. cracks in the electrode, is a very effective way to improve mass transport in fuel cells and shows the potential to be applied in other energy storage and conversion devices. However, there would be manufacturing issue of our process including the morphology distortion during stretching process and adaptability to commercialized MEAs with large area. Hence, proper design of the stretcher machine and initially sprayed region should be further investigated.

## Methods

### Preparation of prism-patterned Nafion membranes

The prism-patterned masters used in this study were prepared by mechanical machining. A plate of stainless steel was machined by using a cutting tool with specific angle. In the process, the height of the prism pattern depended on the cutting depth of the diamond tool. In this study, prism masters with 20 μm and 50 μm periods and the same prism angle (~45°) were used. After preparation of the prism master, a prepolymer resin of perfluoropolyether (PFPE) was dispensed onto the prism master mould. Then, PFPE prism moulds were fabricated by UV replica moulding. The cured PFPE moulds were peeled from the master and cut prior to use. The electrolyte membrane (Nafion 212 membrane) was placed uniformly between a glass substrate and the as-prepared PFPE mould. The sandwiched assembly was compressed at ~95 °C under a hydraulic pressure of 10–20 kg cm^−2^ for 10 min. After cooling the assembly, the prism-patterned Nafion membrane was peeled from the glass substrate and kept in deionized water for 12 h.

### Preparation of prism-patterned MEAs

A catalyst ink was prepared by mixing deionized water, a 5 wt% Nafion ionomer solution, and isopropyl alcohol with carbon-supported catalysts (40 wt% Pt/C, Alfa Aesar, HiSPEC^®^ 4000). The prepared catalyst ink was blended by ultrasonication and sprayed onto the cathode side of the as-prepared prism-patterned Nafion membrane to construct the MEAs. The catalyst Pt loadings were ~0.3 mg cm^−2^ on the cathode sides of the MEAs. The catalyst-coated prism membranes were dried at room temperature (~25 °C) for 12 h. After stretching the catalyst-coated membranes, the catalyst inks were sprayed onto the anode with Pt loadings of ~0.3 mg cm^−2^. Then, Teflon-type gaskets, gas diffusion layers (JNTG-30-A3), and serpentine-type channels were placed onto the cathode and anode without a hot-press process.

### Stretching process for the prism-patterned catalyst-coated membranes

The as-prepared catalyst-coated prism membranes were stretched using a stretching machine (Intron Corp.) to provide uniform strains to the membranes. The upper and lower sides of the MEA were pressed using two mechanical wedge grips with widths of ~5 cm to fix the MEA. The lower side of the MEA was fixed and the upper side of the MEA was moved upward to apply strain to the MEA. The MEA was stretched by varying the applied strain (~0.5 and ~1.0). MEAs were also prepared without the stretching process (i.e. conventional MEAs).

### Physical analysis

The scanning electron microscopy (SEM) images were acquired by using a field emission scanning electron microscope (Carl Zeiss) at an acceleration voltage of 10.0 kV to observe the morphologies of the membrane and electrode layers of the samples. The water behaviour was examined using ESEM (XL-30 FEG). We observed the water condensation behaviour with respect to time based on the relative humidity (RH; about 95%) by maintaining the chamber pressure at ~5.1 Torr and the substrate temperature at ~2 °C.

### FEM analysis

FEM analysis of the stress distribution during the stretching process was performed using the COMSOL Multiphysics software. The behaviours of the elastic and plastic materials were controlled by a governing equation of solid mechanics^33^. Additionally, the plastic property was applied only to the Nafion membrane because the stress applied to the membrane exceeds the elastic limit during the stretching process. Material information for the porous electrode composed of Pt/C, including the elastic modulus and density, was taken from previous studies^9,34^.

### Electrochemical measurements and analysis

The constructed MEAs were incorporated in single cells (CNL Energy). To measure the device performance of a single cell with ~5.0 cm^−2^ active area at 70 °C, humidified O_2_ (or air) and H_2_ gases were supplied to the cathode and anode sides, respectively. The stoichiometric coefficient of H_2_/air was 2.0/9.5. Additionally, the RH values for the anode and cathode gases were both 100%. Electrochemical impedance spectroscopy (EIS, ZENNIUM electrochemical workstation, Zahner) was performed for the single cells at 1.6 A cm^−2^ with an amplitude of 500 mA and at 0.6 V with an amplitude of 5 mV. The frequency range of the measurements was from 0.1 Hz to 100 kHz. The Z-View program (Scribner Associates Incorporation) was used to fit the EIS data. Other experimental conditions, including gas humidification and temperature, were the same as those used for single cell operation at 70 °C with hydrogen/air. Cyclic voltammograms were obtained between 0.05 and 1.20 V at 100 mV s^−1^ to measure the electrochemical active surface of the constructed cathode catalyst layers at room temperature. Humidified N_2_ and H_2_ gases were supplied to the cathode and anode, respectively, and the RH was 100% during the cyclic voltammetry (CV) measurements. The cathode with N_2_ gas served as the working electrode and the anode with H_2_ gas was used as the reference and counter electrodes.

## Electronic supplementary material

Supplementary Video

Supplementary Information
