# Supplementary material for: Guided cracking of electrodes by stretching prism-patterned membrane electrode assemblies for high-performance fuel cells
Source: Sci Rep. 2018 Jan 19;8:1257. doi: 10.1038/s41598-018-19861-6 (PMC5775251; doi:10.1038/s41598-018-19861-6)
Supplement: Supplementary file 2 — Supplementary Information [file 41598_2018_19861_MOESM2_ESM.pdf]

Supplementary Information

Guided cracking of electrodes by stretching prism-patterned membrane electrode assemblies for high-performance fuel cells

Chi-Yeong Ahn[1,2†], Segeun Jang[3,4†], Yong-Hun Cho[5], Jiwoo Choi[3,4], Sungjun Kim[1,2], Sang Moon Kim[6], Yung-Eun Sung[1,2*] and Mansoo Choi[3,4*]*

[1] *Center for Nanoparticle Research, Institute for Basic Science (IBS), Seoul 08826, Korea*

[2] *School of Chemical and Biological Engineering, Seoul National University, Seoul 08826, Korea*

[3] *Global Frontier Center for Multiscale Energy Systems, Seoul National University, Seoul 08826, Korea*

[4] *Department of Mechanical and Aerospace Engineering, Seoul National University, Seoul 08826, Korea*

[5] *Department of Chemical Engineering, Kangwon National University, Samcheok 25913, Korea*

[6] *Department of Mechanical Engineering, Incheon National University, Incheon 22012, Korea*

[†] These authors contributed equally to this work.

* Corresponding authors: mchoi@snu.ac.kr (M. Choi), ysung@snu.ac.kr (Y.-E. Sung), ksm7852@inu.ac.kr (S. M. Kim)

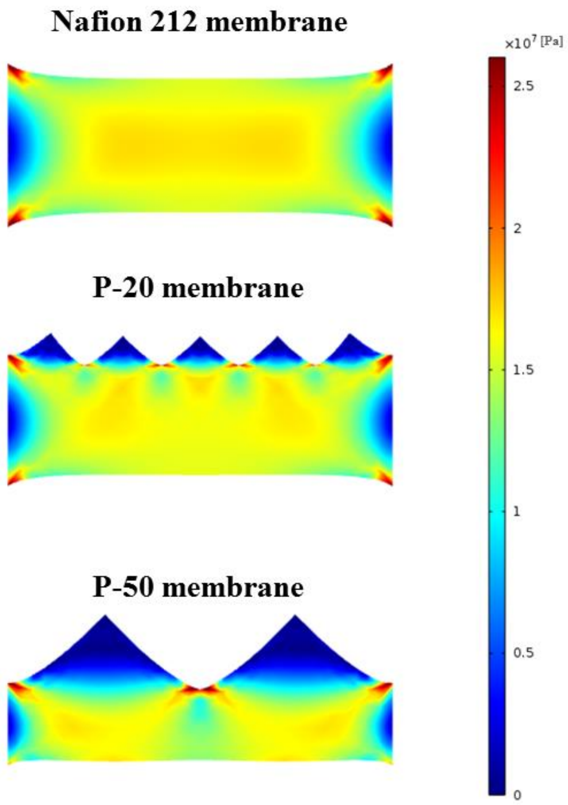

Supplementary Figure S1. Simulation results for the stress distribution on a Nafion 212 membrane and prism-patterned membranes with pitches of 20 μm and 50 μm.

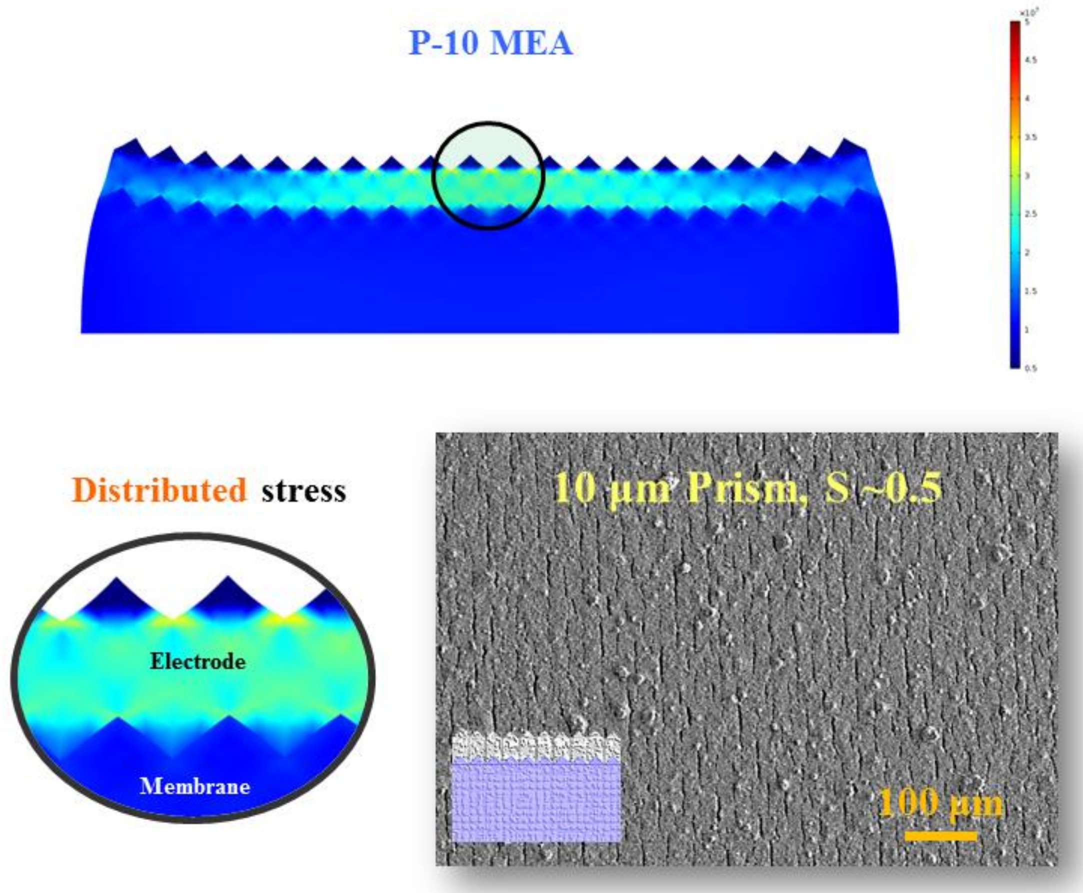

Supplementary Figure S2. Simulation of the stress distribution on a prism-patterned MEA with a pitch of 10 µm and an applied strain of ~0.5, and the corresponding SEM image.

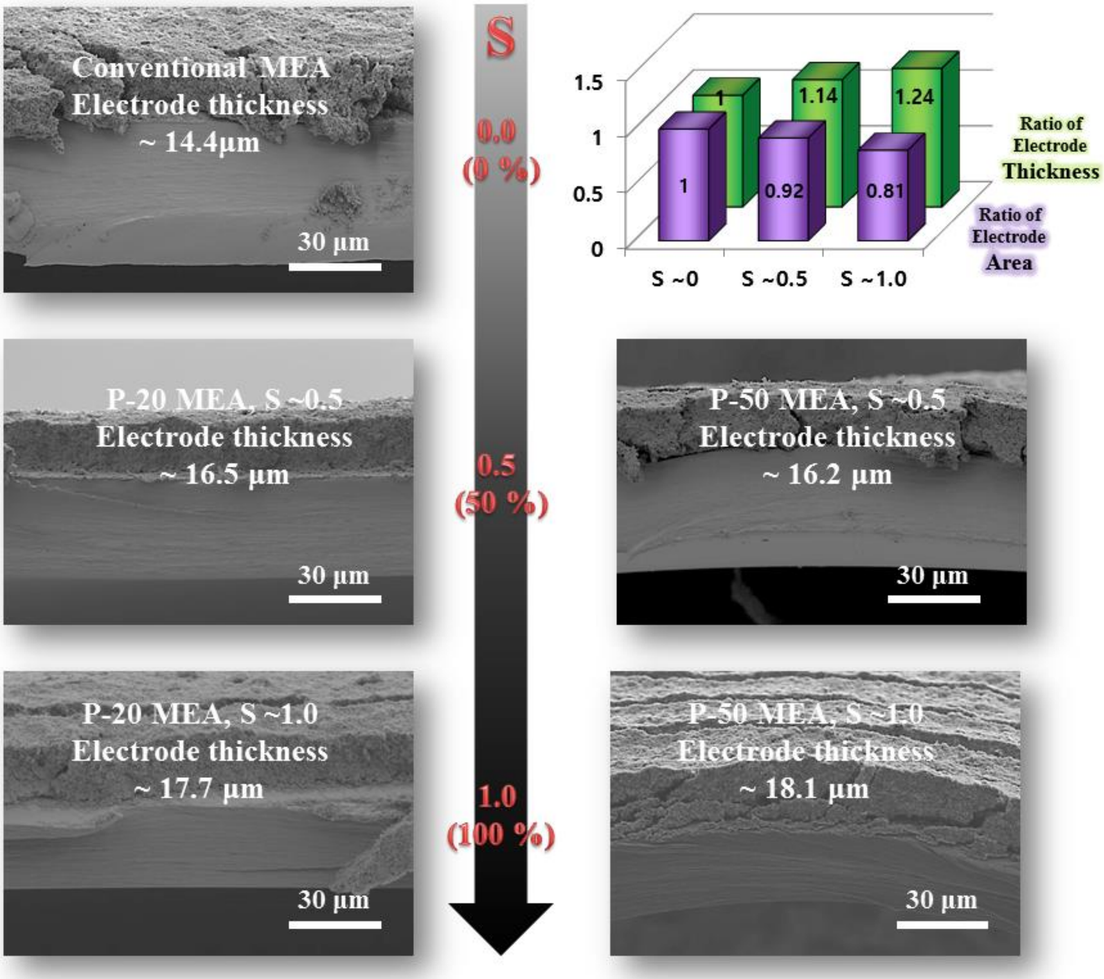

Supplementary Figure S3. Cross-sectional SEM images of the P-20 and P-50 MEAs with $S \approx 0.5$ and 1.0, and a conventional MEA.

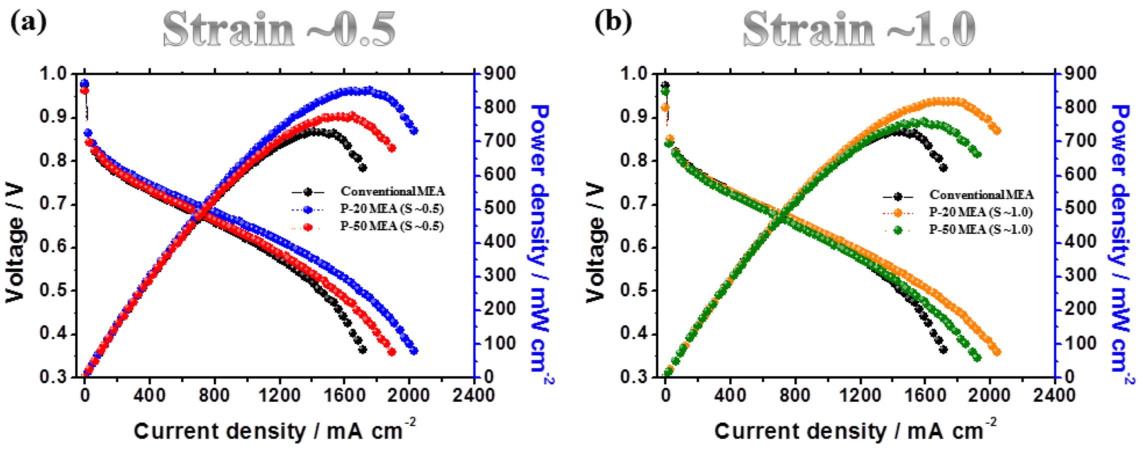

Supplementary Figure S4. Measurements of device performance: (a, b) Polarization curves of a conventional MEA, P-20 MEA, and P-50 MEA with the same applied strain of ~0.5 (a) and ~1.0 (b) under H_2/air.

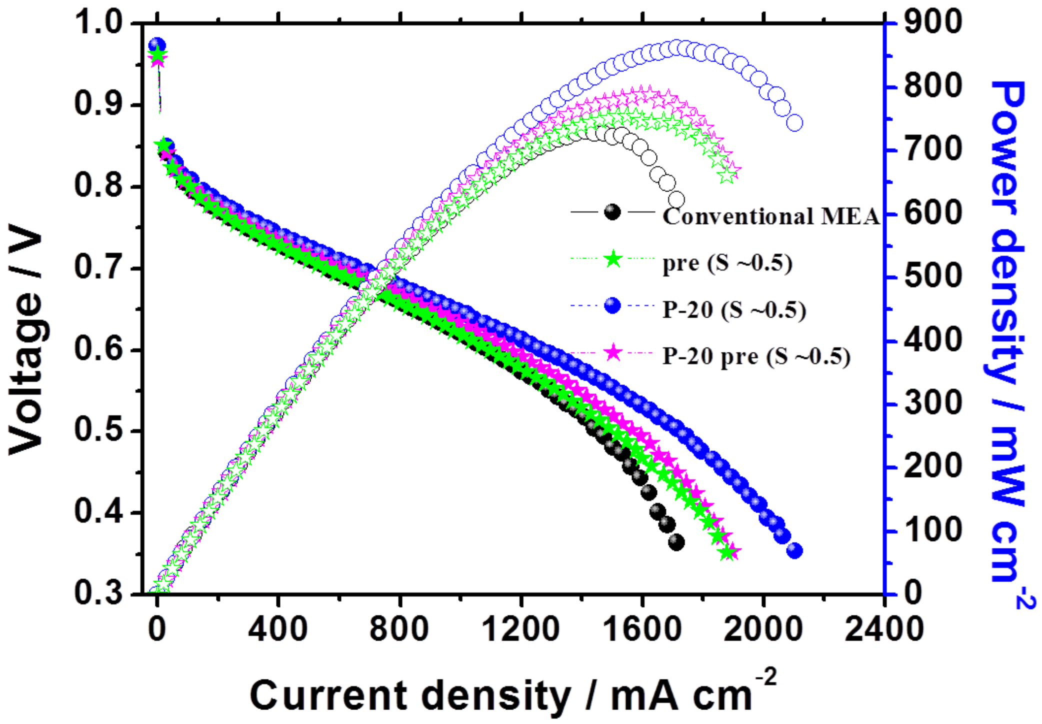

	Current density at 0.6 V (mA cm^{-2})	Max. Power density (mW cm^{-2})
Conventional MEA	1,084	730
Pre (S~0.5)	1,098	755
P-20 (S ~0.5)	1,282	865
P-20 pre (S ~0.5)	1,166	794

Supplementary Figure S5. Polarization curves of conventional MEA, MEA with pre-stretched membrane (S ~0.5), P-20 patterned MEA with pre-stretched membrane (S ~0.5) and P-20 patterned MEA with controlled cracks via stretching MEA (S ~0.5)

Supplementary Table S1. Physical properties of the Nafion membrane.

	Tensile strength, max. [MPa]	Elongation to break [%]
Prism 50 μm	27.61[a)]	260[a)]
Prism 20 μm	27.27[a)]	245[a)]
Nafion 212	29.48[a)]<br>32.00[b)]	291[a)]<br>343[b)]

[a)]Values determined by strain tension tests; [b)]General properties of Nafion® PFSA membrane presented by DuPont™. Please refer to www.fuelcellmarkets.com/content/images/articles/nae201.pdf

Supplementary Table S2. Maximum power densities and current densities at 0.6 V for the prism-patterned MEAs.[a)]

	P-20 MEA		P-50 MEA	
	Current density at 0.6 V [mA cm^{-2}]	Max. power density [mW cm^{-2}]	Current density at 0.6 V [mA cm^{-2}]	Max. power density [mW cm^{-2}]
Strain ~0.5	1,272	863	1,140	781
Strain ~1.0	1,162	821	1,079	764

[a)]Conventional MEA: current density of 1,008 mA cm^{-2} at 0.6 V and max. power density of 730 mW cm^{-2}

Supplementary Table S3. Electrochemical impedance spectroscopy (EIS) fitted data.

	EIS at 0.6 V		EIS at 1.6 A cm^{-2}	
	$R_{membrane}$ [Ω cm^2]	Z_W (Warburg) [Ω cm^2]	$R_{membrane}$ [Ω cm^2]	Z_W (Warburg) [Ω cm^2]
Conventional MEA	0.0631<br>(100%)	0.09565<br>(100%)	0.0660<br>(100%)	0.3702<br>(100%)
P-20 MEA with $S \approx 0.5$	0.0637<br>(101.0%)	0.02026<br>(21.2%)	0.0657<br>(99.5%)	0.0326<br>(8.8%)
P-50 MEA with $S \approx 0.5$	0.0609<br>(96.5%)	0.03817<br>(39.9%)	0.0629<br>(95.3%)	0.0328<br>(8.8%)
